# Schwann Cell Metabolic Activity in Various Short-Term Holding Conditions: Implications for Improved Nerve Graft Viability

**DOI:** 10.1155/2012/742183

**Published:** 2012-01-04

**Authors:** Insa Janssen, Kerstin Reimers, Christina Allmeling, Stella Matthes, Peter M. Vogt, Christine Radtke

**Affiliations:** Department of Plastic, Hand and Reconstructive Surgery, Hannover Medical School, 30625 Hannover, Germany

## Abstract

Strategies for improvement of nerve regeneration and optimal conditions to prevent Schwann cell (SC) loss within a nerve transplant procedure are critical. The purpose of this study was to examine SC viability, which plays an important role in peripheral nerve regeneration, under various incubation conditions up to three hours. To address this issue, Schwann cell metabolic activity was determined using different independent test methods. The following experimental conditions were compared: SCs prepared from nerves were incubated in (1) isotonic saline solution (2) Dulbecco's modified Eagles medium as used for cell culturing, (3) Hannover bioreactor medium, and (4) Leibovitz's medium. SC metabolic activity of excised rat sciatic nerve was determined at 4°C, 18°C, and 37°C over 3 hrs. The results indicate that SC activity was optimized by the usage of Leibovitz's medium or HBRM at 37°C. Greater SC viability at the time of surgical nerve grafting could contribute to improved axonal regeneration and remyelination after nerve transplantation, and thus more successful functional recovery.

## 1. Introduction

Axonal regeneration and remyelination after peripheral nerve injury can be robust with significant functional recovery in contrast to the central nervous system where long white matter tract regeneration is absent or minimal [1]. After peripheral nerve transection, for example, after tumor resection, Wallerian degeneration characterized by macrophage infiltration, axonal membrane digestion, and retraction and proliferation of SCs occurs in the distal nerve segment [2]. The detached SCs from the degenerating axons upregulate the expression of nerve growth factor (NGF) and its low-affinity receptor p75NGFR [3]. For a period of time these SCs are activated [4] and provide trophic support for regeneration. Regeneration occurs from the proximal stump by axonal sprouting and elongation and continues into the distal stump or nerve transplant [5]. The status of a nerve transplant is critical for successful nerve regeneration.

While nerve regeneration through Schwann-cell-enriched basal lamina tubes can reestablish connections with peripheral targets such as skin and muscle, a number of issues, such as navigation of axons across a complex nerve injury site where scarring can occur and appropriate targeting to peripheral end structures are major clinical concerns [6]. Although local endogenous SCs play an important role in regeneration of peripheral nerve, transplantation of additional Schwann cells into a lesion site was shown to assist this regenerative process [7, 8]. Nerve repair combined with transplantation of myelin-forming cell is a relatively simple, rapid, and efficient means of peripheral nerve repair [9]. Moreover, functional nerve regeneration requires not only axonal sprouting and elongation, but also remyelination and appropriate ion channel deployment at the node of Ranvier [8, 9]. The combination of surgical nerve repair and transplantation of peripheral myelin-forming cells has been shown to enhance axonal regeneration and remyelinate demyelinated fibers in experimental models [10] and is currently being investigated in clinical studies [7, 11].

In nerve defect injuries treated with autologous nerve transplantation, the nerve fibers within the transplant contain a high number of Schwann cells which are indirectly transplanted; the viability and activity of these indirectly engrafted Schwann cells may be critical to optimize success of the nerve graft. The importance of the Schwann cells and their basal lamina for axonal regeneration is well established [4, 10, 12]. Loss of viable Schwann cells in a nerve graft results in a reduced neurotrophic support with an attendant reduction in regeneration [13, 14]. Thus, the holding conditions for nerve segments removed for nerve grafting are critical for both SC preservation and potential success of the graft. In the present study, we compared various holding conditions including temperature and medium for short-term preservation (up to 3 hours) followed by determination of Schwann cell viability assessed by two independent test methods after dissociation of excised nerve segments as used for nerve transplantation.

## 2. Materials and Methods

### 2.1. Isolation and Cultivation of Schwann Cells

**Figure 1:**
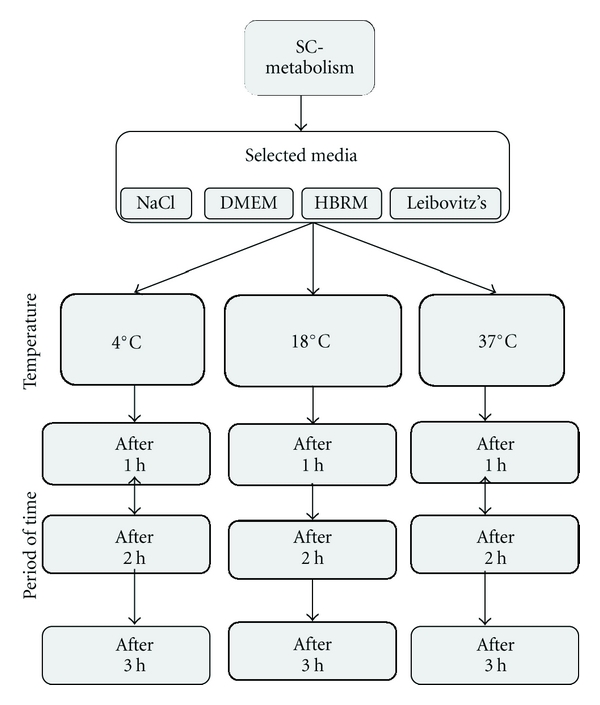
Conditions for testing the metabolic activity of Schwann cells in vitro after incubation in selected media including saline, DMEM with 10% FCS, HBRM, and Leibovitz's medium. Sciatic nerves were incubated in these selected media at 4°C, a room temperature 18°C and 37°C. For each temperature condition, Schwann cell metabolism was determined after 1 hrs, 2 hrs, and 3 hrs.

Experiments were performed in accordance with the German Animal welfare guidelines for the care and use of laboratory animals. The Hannover Medical School and the Nds. Landesamt für Verbraucherschutz und Lebensmittelsicherheit approved all animal protocols. For preparation of adult Schwann cells, adult male Sprague-Dawley rats were deeply anesthetized and sciatic nerves were removed. The sciatic nerves were desheathed, minced, and washed with 10 mL serum-free DMEM low glucose (1 g/L) with L-Glutamine (PAA, Pasching Austria), transferred to a 15 mL tube (TPP, Europe, Switzerland) and washed by centrifugation. For enzymatic dissociation, 15 mg lyophilized Collagenases A and D (Roche, Mannheim, Germany) were dissolved in 10 mL serum-free DMEM; the nerve tissue was incubated at 37°C and 5% CO_2_ for 20 min followed by trituration through a fire-polished siliconized pasteur pipette and washed for three times with DMEM containing 10% FCS. The cells were resuspended and either seeded onto two 25 cm^2^ cell culture flasks (TPP, Europe, Switzerland) coated with Laminin (Engelbreth-Holm-Swarm murine sarcoma basement membrane, Sigma-Aldrich, Steinheim, Germany) for immunocytochemical characterization or plated onto 96-well plates for cell viability assay. The viability of Schwann cells was measured before and after incubation for 1 hr, 2 hrs, and 3 hrs in selected media (see below) at 4°C, 37°C, and 18°C (Figure 1).

### 2.2. Characterization and Quantification of Schwann Cells by Immune Fluorescence

The purity of the prepared Schwann cells was determined by indirect immunofluorescence staining for Schwann cell characteristic marker anti-S100 (polyclonal Rabbit, Dako, Glostrup, Denmark) and visualized with secondary antibody Alexa Fluor 546 (secondary antibody, donkey anti-rabbit, Invitrogen, Karlsruhe, Germany). Nuclei were counterstained with DAPI (Vector Laboratories, CA, USA). Quantification of purity was obtained by counting immunopositive S100 cells in ratio to the number of nuclei. Schwann cell purity was >95% (data not shown).

### 2.3. Measurement of Metabolic Activity

Viability of Schwann cells of sciatic nerves after incubation in selected media was measured by the CellTiter-Blue (CTB) Cell Viability Assay (Promega, Madison, WI, USA), the CellTiter 96 AQueous Nonradioactive Cell Proliferation Assay (Promega, Madison, WI, USA), which indicates cell viability on the basis of cell metabolism. The CTB assay uses the capacity of viable cells reducing the dye resazurin to resorufin, which emits fluorescence at 590 nm. The intensity of fluorescence is proportional to the number of viable cells. As second independent test to verify results, the CellTiter 96 Aqueous Assay contains a tetrazolium compound (MTS; [3-(4,5-dimethylthiazol-2-yl)-5-(3-carboxymethoxyphenyl)-2-(4-sulfophenyl)-2H-tetrazolium, inner salt]) and PMS (phenazine methosulfate) as an electron coupling reagent. Viable cells reduce the MTS reagent into formazan. The absorbance of formazan can be measured at 490 nm and the quantity of absorbance is directly proportional to the number of viable cells.

Selected media for storage were (1) isotonic sodium chloride (NaCl 0.9%, Deltaselect GmbH, Dreieich), (2) DMEM low glucose (1 g/L) supplemented with L-Glutamine (PAA, Pasching, Austria) and 10% FCS (fetal calf serum, Biochrom AG, Berlin, Germany) and 1% Penicillin/Streptomycin (Biochrom AG, Berlin, Germany), (3) Hannover bioreactor medium (HBRM; consisting out of 1000 mL DMEM-F12 (Biochrom AG Berlin, Germany), 10 mL/insulin-transferrin-selenium-A supplement (Gibco), 456 IU Prednisolone, 400 IU Insulin 108 mg/mL Glucagon, 10 *μ*L Soludecortin (10 mg/mL), 5 mL Amphomoronal (Biochrom AG, Berlin, Germany), 20 mL HEPES (Biochrom AG, Berlin, Germany), 50 mg Hyaluronic acid (Sigma Aldrich, Germany), 5 mg/L Penicillin/Streptomycin (Biochrom AG, Berlin, Germany) and (4) Leibovitz's medium (L-15 medium with L-Glutamine mit 25 mM Hepes, PAA, Pasching, Austria). After incubation, Schwann cells were seeded onto 96-well plates (Nunc, NY, USA) with 5000 Schwann cells/well. After 1 hr, 2 hrs, and 3 hrs, 20 *μ*L of the CTB-reagent was added to the dissociated cells and measurements were performed after incubation according to the suppliers' specifications. All measurements were repeated at three independent times. The background fluorescence or absorbance of microtiter plates with tested medium and lacking cells was subtracted from these data.

### 2.4. Statistics

All statistical analysis of data was performed using SigmaStat software (SPSS, Chicago, IL, USA). All data were evaluated using one-way ANOVA and post hoc analysis using the Student-Newman-Keuls method. All data are expressed as means ± SE.

## 3. Results

Standardized 2 cm segments of rat sciatic nerve were removed and Schwann cells were prepared for culture. Schwann cell viability was determined after incubation in either saline, DMEM, HBRM, or Leibovitz's solution (*n* = 3 per group) for 0 hr, 1 hrs, 2 hrs, and 3 hrs. For each medium condition and time point, metabolic activity was studied at three separate temperatures: 4°C, room temperature 18°C, and 37°C (Figure 1). Cell viability was determined by two independent methods: the CTB and MTS test.

### 3.1. Metabolic Activity of Schwann Cells at 4°C

**Figure 2:**
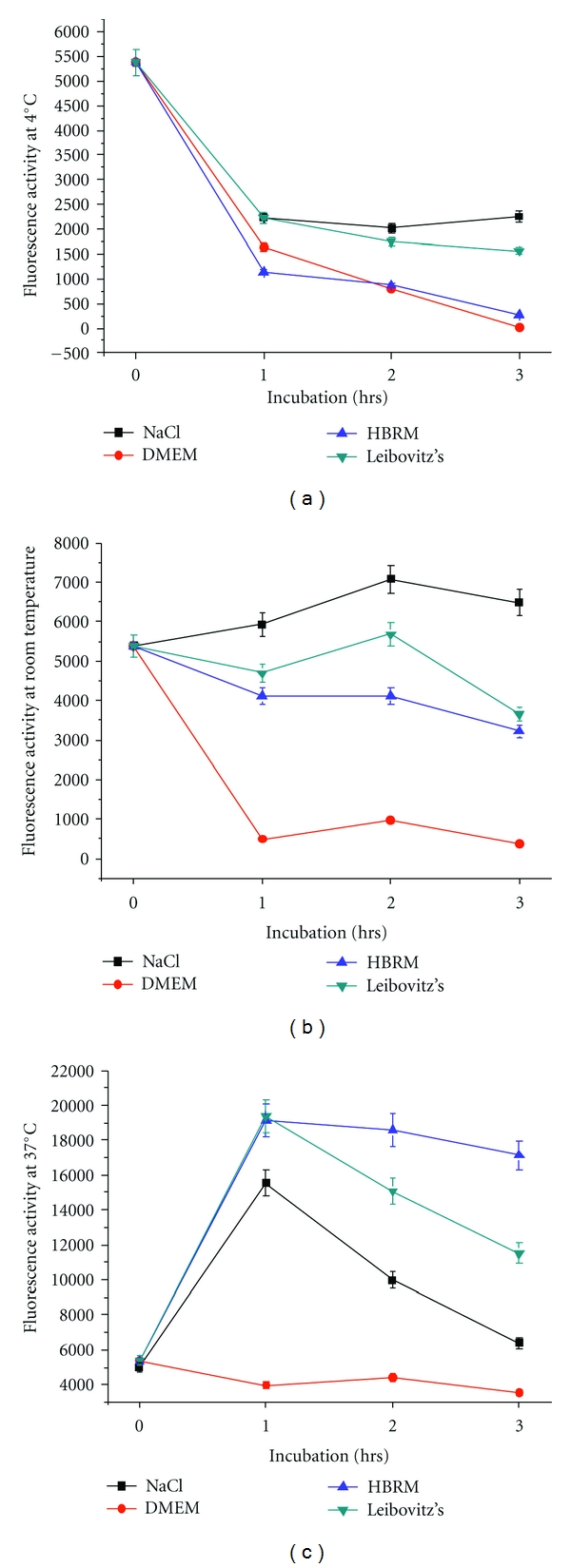
This Figure and respectively, Figure 3: Schwann cell metabolism indicated as fluorescence activity at 4°C (a), room temperature (b), and at 37°C (in (c)) incubated in the described media. Cell viability was measured by reduction of resazurin to resofurin by CellTiter-Blue (CTB) Cell Viability Assay. Most reduced cell viability was observed after 3-hour incubation of Schwann cells in DMEM at 4°C. Optimal cell viability with increase of Schwann cell metabolism can be seen after incubation in HBRM at 37°C. *P* < 0.05.

**Figure 3:**
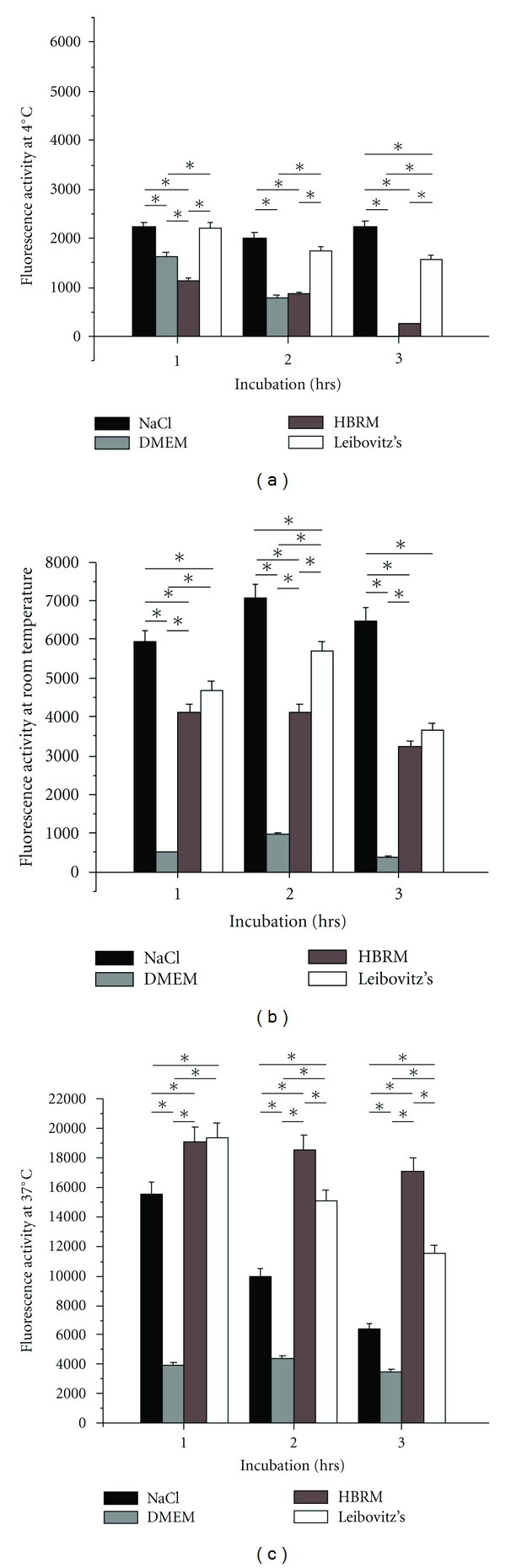


Metabolic activity was studied upon dissociation of the Schwann cells of rat sciatic nerves after incubation in the selected four media conditions and measured by CTB (Figure 2 for line chart and Figure 3 for bar chart). Schwann cell metabolic activity incubated at 4°C (Figure 2(a) and Figure 3(a), resp.) was decreased at 1 hr and remained decreased in the 2 hrs and 3 hrs groups. In general, at 4°C the metabolic activity was the lowest for the DMEM condition. Thus, Schwann cell metabolic activity in culture was reduced with time with all three media significantly. The cell viability was more than reduced to half within the first hour in all four experimental groups. The greatest Schwann cell viability in this set of experiments at 4°C could be observed in saline where cell metabolic activity was significantly higher than in DMEM and HBRM. The lowest viability was obtained at 4°C in DMEM with about 70% reduction in metabolic activity within the first hour followed by a reduction, nearly complete loss of metabolic activity, of 99.7% to 0.3% after 3 hours. Cell viability was examined by MTS.

### 3.2. Metabolic Activity of Schwann Cells at Room Temperature

At room temperature (Figure 2(b) and Figure 3(b), resp.), metabolic activity of cultured Schwann cells determined by CTB decreased for the DMEM and HBRM conditions, nominally was unchanged for the Leibovitz's medium and marginally increased in saline over time (Figures 2(b) and 3(b)). The reduction in metabolic activity was observed in DMEM with most loss of cell viability within the first hour of more than 90%. An increase of cell metabolic activity (132%) with incubation in saline occurred within the first 2 hours of cell activity followed by a continued reduction of approximately 10% within the third hour. The poorest conditions were obtained at room temperature with DMEM. Thus, the best Schwann cell activity at room temperature could be seen in the saline group over the three-hour period with increase of cell metabolism.

### 3.3. Metabolic Activity for Schwann Cells Incubated at 37°C

Metabolic activity for Schwann cells incubated at 37°C and measured by CTB increased for those incubated in HBRM, Leibovitz and saline (Figure 2(c) and Figure 3(c), respectively). Activity of Schwann cells in HBRM increased nearly 4-fold within the first hour followed by a slight reduction at the 2-hour time point, but still showing an increase in cell metabolism of 318% at the 3-hour time point. Incubation in Leibovitz's medium showed a similar 4-fold increase of Schwann cell metabolism within the first hour followed by continued decrease within the next 2 hours of observation period. After the three-hour period, there is still an increase of activity of 100% noticeable. Schwann cells metabolism after incubation in saline showed a 3-fold increase within the first hour resulting in an increase of activity of 120% after 3 hours. Schwann metabolism preincubated in DMEM did not show an increase in metabolic activity. The increase in metabolic activity for these HBRM, saline, and Leibovitz's medium peaked at 1 hr after nerve incubation at 37°C. Metabolic activity was reduced for all three conditions at 2 hrs and 3 hrs, but remained higher than at 0 hr. For nerves, the metabolic activity remained continuously high when incubated with HBRM at 2 and 3 hrs, indicating a broader time window for postincubation Schwann cell metabolic activity than the other 3 media.

### 3.4. Metabolic Activity of Schwann Cells Measured by MTS at 4°C, Room Temperature, and 37°C

**Figure 4:**
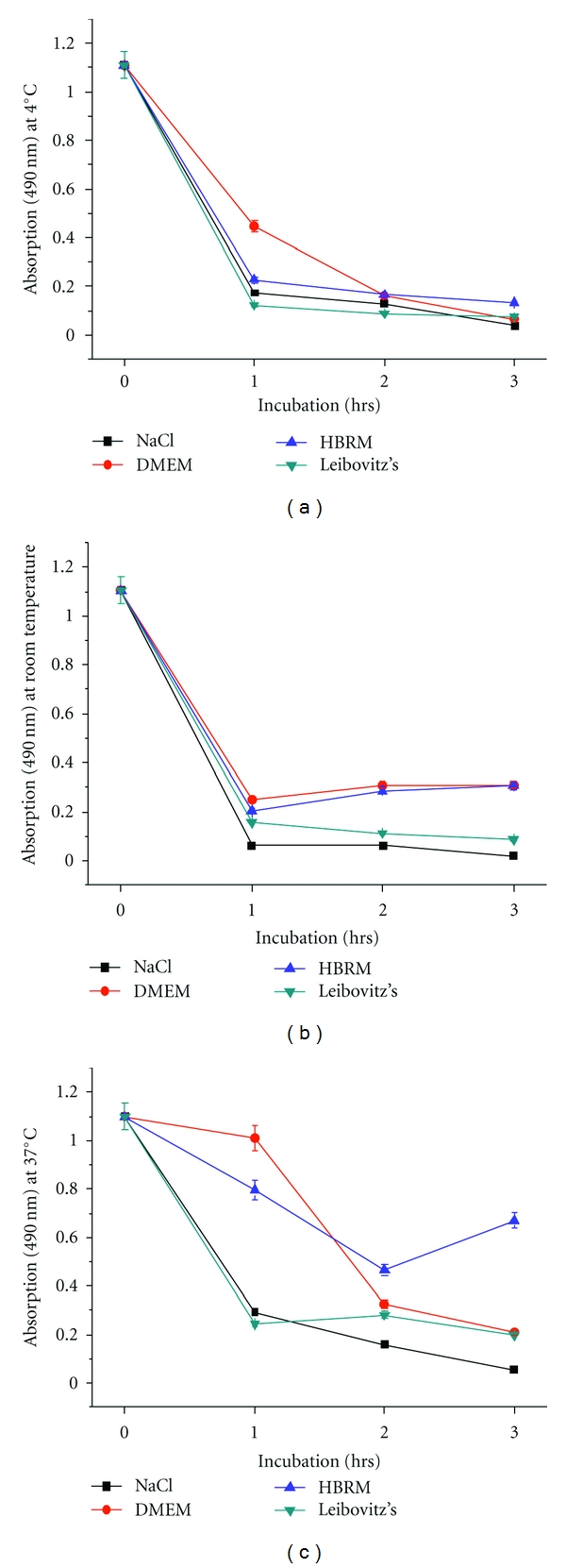
Schwann cell metabolism indicated as absorbance measured with MTS at 4°C (a), room temperature (b), and at 37°C (c) incubated in the described media. At 4°C all conditions resulted after 3 hours in massive reduction of cell viability. At room temperature incubation in HRBM and DMEM showed slightly better results than NaCl and Leibovitz's. Optimal cell viability with increase of Schwann cell metabolism can be seen after incubation in HBRM at 37°C as observed by independent determination of cell viability by CTB in Figure 2. *P* < 0.05.

Additionally, Schwann cell metabolism was also assessed as absorbance measured with MTS at 4°C (Figure 4(a)), room temperature (Figure 4(b)), and at 37°C (Figure 4(c)) incubated in the described media. At 4°C, all conditions resulted after 3 hours in massive reduction of cell viability. At room temperature, incubation in HRBM and DMEM showed slightly better results than NaCl and Leibovitz. Optimal cell viability with increase of Schwann cell metabolism can be seen after incubation in HBRM at 37°C as observed by determination of cell viability by reducing resazurin to resofurin with CTB in Figure 2.

 Thus, Schwann cell metabolic activity over time in culture from ex vivo nerves incubated in one of four media was generally reduced at 4°C and room temperature incubation temperatures over a time window for three hours as measured by CTB and MTS independently. Most reduction in cell viability was observed within the first hour of incubation in both tests of more than 50% in all media at 4°C. For incubation at 37°C, Schwann cell metabolic activity was increased for nerve segments incubated in HBRM, Leibovitz, and saline over the observation period of three hours. But, there was no increase at 37°C in Schwann cell metabolic activity incubated in DMEM. While Schwann cell metabolic activity peaked at the 1 hr nerve incubation time point and declined for Leibovitz's and saline, the activity remained high even at the 2- and 3-hour time points. Most reduced cell viability was observed after 3-hour incubation of Schwann cells in DMEM at 4°C. Optimal cell viability with increase of Schwann cell metabolism can be seen after incubation in HBRM at 37°C. This could be verified by the results obtained with the MTS at 37°C.

## 4. Discussion

In this study, we demonstrated that Schwann cell metabolic activity is dependent on several factors including temperature, holding media, incubation, and holding time. The greatest increase in metabolic activity of all these groups was observed at 37°C over a period of three hours. Here, a significant increase in metabolic activity could be observed in Leibovitz's medium and HBRM with a 3-fold increase after incubation of 3 hours in HBRM. Saline resulted in moderate increase of 120% in comparison to the other groups at 37°C.

 Observing just 1 hour of incubation period at 37°C, best results were observed in correspondence to 3 three hours in saline, Leibovitz's medium, and HBRM with maximum activity with a 4-fold increase after 1 hour in both conditions. Most decrease in cell metabolic activity was observed at nerves incubated at 4°C in all 4 experimental groups. Cell metabolism was reduced to half after 3 hours in the saline group and was close to zero metabolic activity in the DMEM group. The DMEM group resulted in all three temperature conditions in most reduction in cell metabolism activity. It is important to point out that our data demonstrated that incubation in the commonly used, the culture medium of Schwann consisting of DMEM with 10% serum did not result at any time point, and temperature in an increase, but instead in a significant decrease in cell metabolism activity within the three hour observation period. Thus, DMEM is suboptimal as a medium for preservation of Schwann cell metabolism and alternatives should be considered.

 Schwann cells play an important role in peripheral nerve regeneration; and poor survival of Schwann cells is thought to influence the outcome of nerve transplantation [15]. Reduced number of Schwann cells after suboptimal storage conditions of donor nerve removed for autologous cell transplantation, for example, in traumatic nerve defect injuries or after wide tumor resection, could lead to a reduction in growth factor production, less axonal guidance of elongating axons resulting in increased sprouting, and failure to connect to peripheral targets or even painful neuroma formation [11].

For improvement of peripheral nerve regeneration, it was shown that additional transplantation of Schwann cells leads to more directed growth of regenerating axons and a significant better functional result with a greater nerve conduction velocity after crush lesion and after microsurgical repair [8–10]. The survival of Schwann cells could be experimentally improved by overexpression of polysialic acid [15] or addition of FK506 [16, 17]. Moreover, pre-degeneration of nerve grafts resulted in improvement of nerve regeneration based on the activation of Schwann cells [17–20]. The efficacy of 2–7 days of 14-day pre-degeneration in DMEM of rat Schwann cell culture was described in a recent study by Kraus et al. (2010) [21].

Moreover, further experimental studies demonstrated the importance of seeded Schwann cells to improve the efficacy of acellular nerve grafts [22] or artificial nerve guidance channels [23, 24] to improve effectiveness of nerve repair. Addition of Schwann cells to muscle as nerve guidance in peripheral nerve defect injuries resulted in significant better functional and histological results than without cell supplement indicating the important role of Schwann cells for enhancement of axonal regeneration and remyelination after injury. Schwann cell-seeded bioartificial nerve conduits are subjects of several ongoing studies to improve functional results after nerve repair and to bridge extended nerve defects [24, 25]. Thus, incubation for optimal Schwann cell metabolic activity of donor nerve or of Schwann cell suspension for direct transplantation is essential and underlines the importance of our presented data. To verify our observed results, we performed independent test methods including using the CTB and MTS assay for comparison of cell metabolism according to previous reports [26, 27]. We archived similar results with all test methods demonstrating optimal cell viability of Schwann cell metabolism after 3-hour incubation in HBRM at 37°C.

In contrast to previous reports, we directly investigated Schwann cell viability which has a critical influence on the histological and functional results after nerve repair and nerve transplants after dissociation in a short period of time of three hours which is clinical relevant. Previous reports examined Schwann cell proliferation after 2 days and 3 days of culture [28]. Here, progressive increase of proliferation over 3 days could be observed. Successful storage of peripheral nerve before transplantation after this time could be demonstrated in an experimental study in rats by using polyphenol to reduce oxidative stress [29]. In this study, nerve segments were kept for up to 30 days in polyphenol solution followed by determination of nerve viability by calcein-AM/ethidium homodimer staining. In another study, storage in polyphenol solution was compared to conventional University of Wisconsin solution for long-term peripheral nerve banking [30].

## 5. Conclusion

The results demonstrated the enhancement of Schwann cell metabolism and optimal Schwann cell viability after incubation at 37°C with usage of either Leibovitz or HBRM up to three hours. The commonly used saline solution at room temperature demonstrated a slight increase in cell metabolism at 37°C. To obtain optimal results after nerve transplantation, removed nerves should be stored in the described conditions with usage of Leibovitz's medium or HBRM in 37°C upon transplantation. Additional experimental studies will determine if the presented results in vitro could be confirmed in vivo. With regard to clinical application, increased SC viability in autologous nerve transplants could result in enhanced axonal regeneration and remyelination leading to improved functional outcome after nerve grafting.

## Abbreviations

SC: Schwann cell
HBRM: Hannover bioreactor medium
CTB: Cell Titer-Blue Cell Viability Assay
DAPI: 4′, 6-diamidino-2-phenylindol
DMEM: Dulbecco's modified Eagles'medium
EDTA: Ethylenediamine tetraacetic acid
FCS: Fetal calf serum
HEPES: 2-(4-(2-Hydroxyethyl)-1-piperazinyl)-ethansulfonsäure
MTS: Tetrazolium salt ([3-(4,5-dimethylthiazol-2-yl)-5-(3-carboxymethoxyphenyl)-2-(4-sulfophenyl)-2H-tetrazolium, inner salt])
NaCl 0.9%: Isotonic 0.9% sodium chloride
NGF: Nerve growth factor
PBS: Phosphate buffered saline solution
PMS: Phenazine methosulfate
p75NGFR: Low affinity nerve growth factor receptor
RT: Room temperature (18°C).
