# Corrigendum to “Schwann Cell Metabolic Activity in Various Short-Term Holding Conditions: Implications for Improved Nerve Graft Viability”

**DOI:** 10.1155/2024/9870507

**Published:** 2024-12-26

**Authors:** Insa Janssen, Kerstin Reimers, Christina Allmeling, Stella Matthes, Peter M. Vogt, Christine Radtke

**Affiliations:** ^1^Department of Plastic, Hand and Reconstructive Surgery, Hannover Medical School, Hannover 30625, Germany; ^2^Department of Neurosurgery, University Hospital Geneva, Geneva 1205, Switzerland; ^3^University Department of Plastic, Reconstructive and Aesthetic Surgery Vienna, Vienna 1090, Austria

In the article titled “Schwann Cell Metabolic Activity in Various Short-Term Holding Conditions: Implications for Improved Nerve Graft Viability” [1], the authors identified a repetitive error concerning the description of the methods. The term “nerve” was used several times and it should have been Schwann cells derived from nerve. The authors would like to emphasize that Schwann cells first were isolated from sciatic nerves and then cultured. Schwann cells in passage 12 were exposed to the different and modified storage conditions in accordance to the clinical procedure of nerve harvesting.

The relevant text passages should be corrected as follows:

Page 2, lines 10–13: “(…) followed by determination of Schwann cell viability assessed by two independent test methods after dissociation of excised nerve segments as used for nerve transplantation” should be corrected to “(…). Schwann cells from excised nerves were cultured followed by determination of cell viability assessed by two independent test methods.”

In the legend of Figure 1 (Page 2), in Section 3.3 (Page 3, lines 36 and 38), in the Section 3.4 (Page 4, lines 6 and 10) and in Section 4 (Page 5, line 10), the term “nerve” should be corrected to “Schwann cells from excised nerves.”
